# NRAS^Q61K^ mutated primary leptomeningeal melanoma in a child: case presentation and discussion on clinical and diagnostic implications

**DOI:** 10.1186/s12885-016-2556-y

**Published:** 2016-07-20

**Authors:** Giulia Angelino, Maria Debora De Pasquale, Luigi De Sio, Annalisa Serra, Luca Massimi, Rita De Vito, Antonio Marrazzo, Laura Lancella, Andrea Carai, Manila Antonelli, Felice Giangaspero, Marco Gessi, Laura Menchini, Laura Scarciolla, Daniela Longo, Angela Mastronuzzi

**Affiliations:** University-Hospital Pediatric Department, Bambino Gesù Children’s Hospital IRCCS and University of Rome Tor Vergata School of Medicine, Rome, Italy; Department of Hematology, Oncology and Stem Cell Transplantation, Bambino Gesù Children’s Hospital IRCCS, Piazza Sant’Onofrio 4, 00165 Rome, Italy; Department of Pediatric Neurosurgery, Catholic University of the Sacred Heart, A. Gemelli University Polyclinic, Rome, Italy; Unit of Pathology, Bambino Gesù Children’s Hospital IRCCS, Rome, Italy; Unit of Infectious Disease, Department of Pediatrics, Bambino Gesù Children’s Hospital IRCCS, Rome, Italy; Unit of Neurosurgery, Bambino Gesù Children’s Hospital IRCCS, Rome, Italy; Department of Radiological, Oncological and Anatomo-Pathological Sciences, Sapienza University, Rome, Italy; Neuromed IRCCS, Pozzilli, Isernia Italy; Institute of Neuropathology, University of Bonn Medical Center, Bonn, Germany; Department of Diagnostic Imaging, Unit of Neuroradiology, Bambino Gesù Children’s Hospital IRCCS, Rome, Italy

**Keywords:** Primary leptomeningeal melanoma, Tuberculous meningitis, NRAS ^Q61K^ mutation, *NRAS* inhibitors, Children

## Abstract

**Background:**

Primary melanocytic neoplasms are rare in the pediatric age. Among them, the pattern of neoplastic meningitis represents a peculiar diagnostic challenge since neuroradiological features may be subtle and cerebrospinal fluid analysis may not be informative. Clinical misdiagnosis of neoplastic meningitis with tuberculous meningitis has been described in few pediatric cases, leading to a significant delay in appropriate management of patients. We describe the case of a child with primary leptomeningeal melanoma (LMM) that was initially misdiagnosed with tuberculous meningitis. We review the clinical and molecular aspects of LMM and discuss on clinical and diagnostic implications.

**Case presentation:**

A 27-month-old girl with a 1-week history of vomiting with mild intermittent strabismus underwent Magnetic Resonance Imaging, showing diffuse brainstem and spinal leptomeningeal enhancement. Cerebrospinal fluid analysis was unremarkable. Antitubercular treatment was started without any improvement. A spinal intradural biopsy was suggestive for primary leptomeningeal melanomatosis. Chemotherapy was started, but general clinical conditions progressively worsened and patient died 11 months after diagnosis. Molecular investigations were performed post-mortem on tumor tissue and revealed absence of BRAF^V600E^, GNAQ^Q209^ and GNA11^Q209^ mutations but the presence of a NRAS^Q61K^ mutation.

**Conclusions:**

Our case adds some information to the limited experience of the literature, confirming the presence of the NRAS^Q61K^ mutation in children with melanomatosis. To our knowledge, this is the first case of leptomeningeal melanocytic neoplasms (LMN) without associated skin lesions to harbor this mutation. Isolated LMN presentation might be insidious, mimicking tuberculous meningitis, and should be suspected if no definite diagnosis is possible or if antitubercular treatment does not result in dramatic clinical improvement. Leptomeningeal biopsy should be considered, not only to confirm diagnosis of LMN but also to study molecular profile. Further molecular profiling and preclinical models will be pivotal in testing combination of target therapy to treat this challenging disease.

## Background

Primary melanocytic neoplasms are rare in the pediatric age and may present with a wide spectrum of clinical and pathological features [1]. Among them, the pattern of neoplastic meningitis represents a peculiar diagnostic challenge since the neuroradiological features may be subtle and cerebrospinal fluid (CSF) analysis may not be informative [1]. Clinical misdiagnosis of neoplastic meningitis with tuberculous meningitis has been described in few pediatric cases, leading to a significant delay in appropriate management of patients (Table 1) [2–7].

**Table 1:** Pediatric case reports of primary leptomeningeal melanoma and neoplastic meningitis mimicking tuberculous meningitis

Reference	Makin, 1999	Nicolaides, 1995	Selcuk, 2008	Demir, 2010	Kosker, 2014	Erdogan, 2014	Our patient
Diagnosis	Primitive leptomeningeal melanoma	Primitive leptomeningeal melanoma	Atypical Teratoid Rhabdoid Tumor	Spinal low-grade neoplasm	Primary diffuse leptomeningeal gliomatosis	Primary spinal leptomeningeal gliomatosis	Primitive leptomeningeal melanoma
Age and Sex	5,5 years, Male	5 years, Male	6 years, Female	8 years, Female	3 years, male	3 years, male	2 years, Female
Onset signs and symptoms	13-week history of headaches, vomiting, and weight loss followed by acute deterioration of conscious level	3-month history of vomiting, anorexia, and weight loss, 1-month history of headaches and pyrexia, acute deterioration of conscious level	2-months history of confusion, headache, vomiting, aphasia, and right hemiparesis	History of headache, nausea, fever, and vomiting, followed by double vision	3-month history of strabismus and 1-week history of headache and restlessness	Deviation of left eye, weakness, lack of appetite, headache and behavioral change	1-week history of vomiting
Imaging at onset	CT: diffuse meningeal enhancement	CT: diffuse meningeal enhancement	MRI: marked leptomeningeal involvement and basal meningitis	MRI: communicating hydrocephalus, diffuse leptomeningeal enhancement at basal cisterns	MRI: leptomeningeal infiltration, prominent around the Sylvian fissure and at the level of the basal cisterns	MRI: diffuse leptomeningeal enhancement, predominantly involving the basal cisterns and hydrocephalus	MRI: enhancement of the cervical and basal meninges and cranial nerves, in addition to a small focal enhancement anterior to the pons
CSF analysis at onset	- Protein 1.5 g/dL - Glucose 0.7 mmol/L - No cells	- Protein 1.5 g/L - Glucose 0.5 mmol/L (serum glucose 5 mmol/L) - Leukocytes 36/mm^3^	- Protein 40.8 mg/dL - Glucose 36 mg/dL (serum glucose 136 mg/dL) - Lymphocytes 350/mm^3^	- Protein 242 mg/dL - Glucose 74 mg/dL (serum glucose 116 mg/dL) - 10 × 5 cells (60 % lymphocyte, 40 % PMNL)	- Protein 9.2 mg/dL - Glucose 102 mg/dL (serum glucose 136 mg/dL) - Leukocytes 470/mm3 (90 % lymphocyte, 10 % PMNL)	- Protein elevated - Glucose normal	- Protein 62 mg/dL - Glucose 83 mg/dL (serum glucose 133 mg/dL) - Leukocytes 2/mm3
CSF cyto-morphological examination	ND	- 1^st^ sample: negative - 2^nd^ sample: positive for malignant cells	Negative	ND	Negative	Negative	- 1^st^ sample: negative - 2^rd^ sample: positive for malignant cells
Time delay between onset of symptoms and definitive diagnosis	3 months	3 months	UNK	4 months	10 months	4 months	10 weeks
Chemotherapy	Vincristine, carboplatin, and etoposide	Chemotherapy according to local protocol (not specified)	Not done (parent’s refusal)	Cisplatin and etoposide; radiotherapy	Vincristine, carboplatin, and etoposide; (parents refused radiotherapy)	Vincristine and carboplatin	Temozolomide, cis-platinum, vindesina and peginterferon alfa-2b; radiotherapy
Outcome	Dead 6 months after diagnosis	Unknown	Dead 3 months after onset	Alive after 19 months follow-up	Alive after 18 months follow-up	Unknown	Dead 11 months after diagnosis

*CSF*, cerebrospinal fluid, *CT* computed tomography, *MRI* magnetic resonance imaging, *ND* not done, *UNK* unknown, *PMNL* polymorphonuclear leukocytes

We describe the case of a child with primary leptomeningeal melanoma (LMM) that was initially misdiagnosed with tuberculous meningitis. We review clinical and molecular aspects of LMM and discuss clinical and diagnostic implications.

## Case presentation

**Fig. 1:**
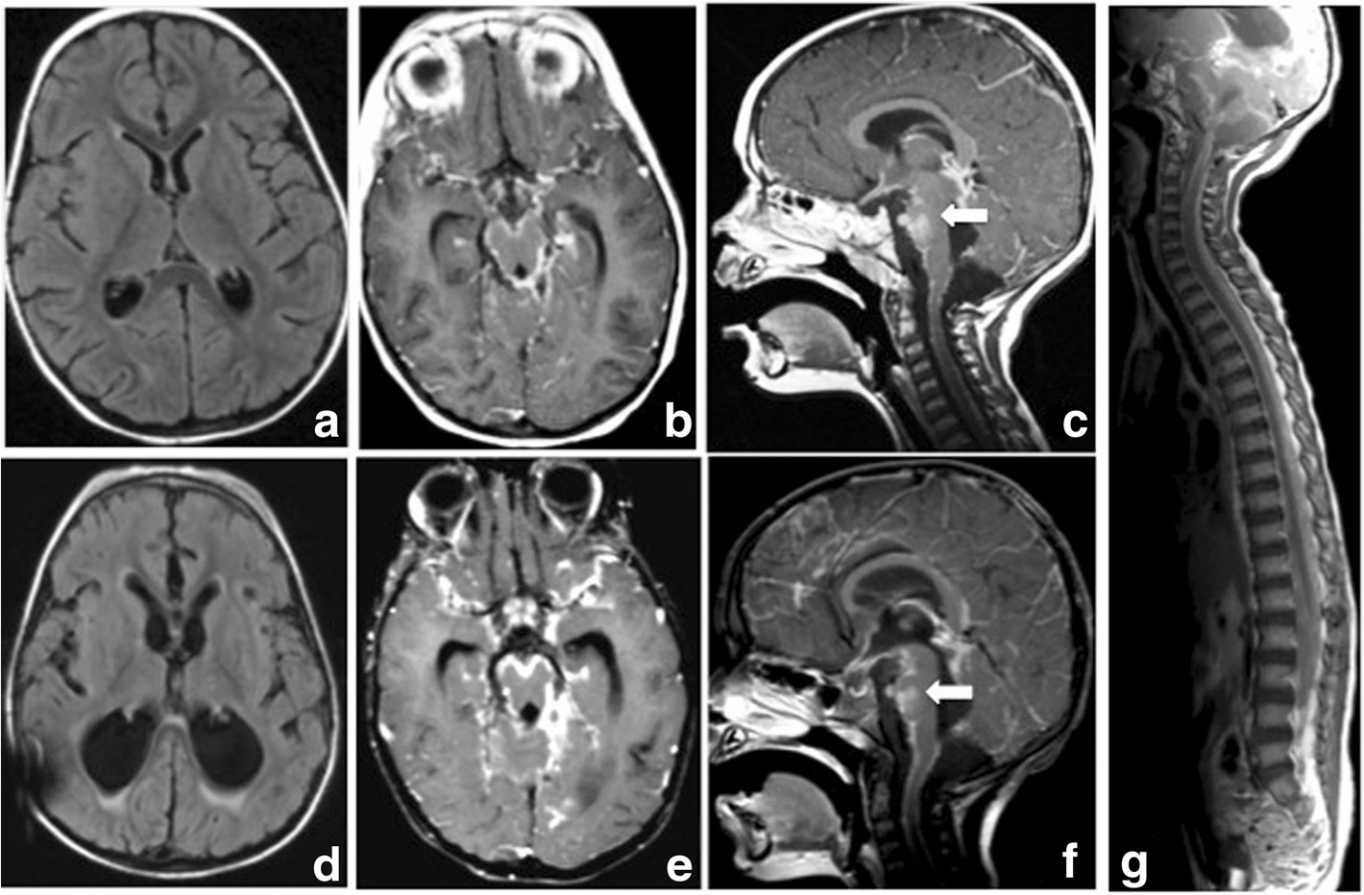
Clinical onset MRI (**a**, **b**, **c**). Follow-up MRI (**d**, **e**, **f**, **g**). T1 axial basal image (**a**): no evidence of LMM’s typical hyperintensities. T1 Contrast enhancement images (**b**, **c**): intense base and peri-spinal leptomeningeal enhancement and nodular pontine enhancing lesion (white arrow); (**e**, **f**, **g**) increase of enhancing lesions. T1 axial (**a**, **d**): progressive hydrocephalus

**Fig. 2:**
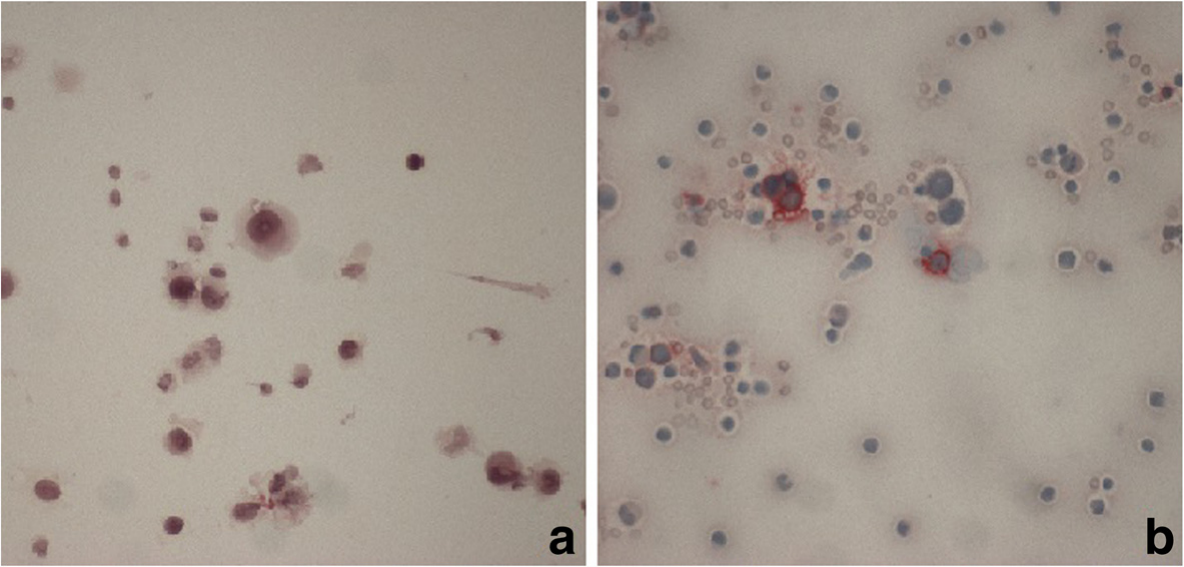
Cyto-morphological examination of CSF. May-Grünwald-Giemsa staining shows numerous polymorphic cells with large cytoplasm and prominent nucleoli (**a**). On immunohistochemical profile cells are positive for S100 (**b**)

**Fig. 3:**
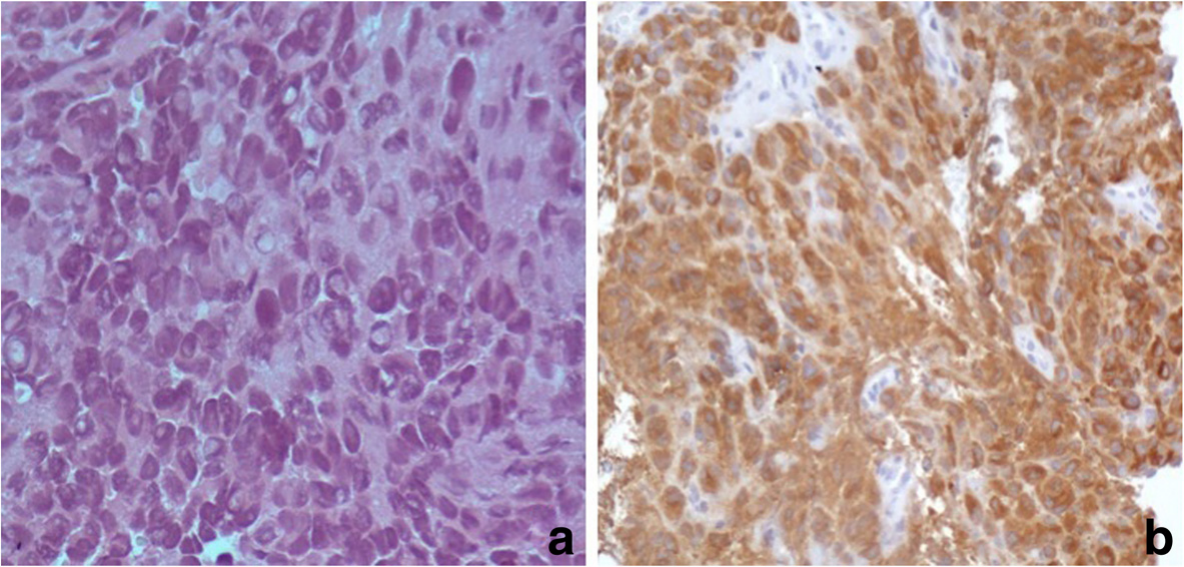
Histological examination of tumor biopsy. Neoplasm is composed of pleomorphic cells with vescicular nuclei, eosinophilic nuclear pseudoinclusion and moderate cytoplasm (**a**). Immunoistochemistry shows intense positivity for MelanA (**b**)

A 27- month-old girl was referred to Bambino Gesù Children's Hospital in 2009 after a 1-week history of vomiting associated to mild intermittent strabismus. Ophthalmologic evaluation revealed bilateral papilledema. Magnetic Resonance Imaging (MRI) showed diffuse brainstem and spinal leptomeningeal enhancement (Fig. 1a–c). CSF analysis was unremarkable. Tuberculosis (TB) was not confirmed by a complete work-up. Nonetheless, antitubercular treatment was started based on the MRI findings. After 10 days the patient was transferred to the Intensive Care Unit for a salt wasting syndrome. A new MRI demonstrated hydrocephalus (Fig. 1d) and progression of leptomeningeal enhancement (Fig. 1e–g). A new CSF examination was done and showed neoplastic cells with large cytoplasm and prominent nucleoli (Fig. 2) positive for S100. Therefore, antitubercular therapy was discontinued and a ventriculoperitoneal shunt was placed because of the progression of neurological symptoms. Moreover, a spinal intradural biopsy was performed: histological examination showed pleomorphic cells with vesicular nuclei, eosinophilic nuclear pseudoinclusion and moderate cytoplasm (Fig. 3). Immunohistochemistry showed intense positivity for MelanA, suggesting the diagnosis of primary leptomeningeal melanomatosis. No signs of cutaneous melanosis were observed. Chemotherapy was started, including temozolomide, cis-platinum, vindesine and peg-interferon alfa-2b. MRI was obtained every two months showing stable disease until the sixth course of chemotherapy when progression was found. At that time, radiation was associated to peg-interferon alfa-2b but the tumor rapidly spread to chest and abdomen. General clinical conditions progressively worsened and patient died 11 months after diagnosis. Molecular investigations were performed post-mortem on tumor tissue and revealed absence of BRAF^V600E^, GNAQ^Q209^ and GNA11^Q209^ mutations but the presence of a NRAS^Q61K^ mutation.

## Conclusions

Primary leptomeningeal melanocytic neoplasms (LMN) can be focal (melanomas) or diffuse (melanomatosis) [8]. Since the first description by Virchow in 1859 [9], primary LMM has been reported in few hundreds of patients, mainly adults, with peak incidence in the fourth decade of life [2, 10, 11]. Pediatric experience is extremely limited, accounting for about 0.1 % of central nervous system tumors, this affecting the diagnostic approach and clinical management of patients. During embryogenesis, melanocytic precursors spread from the neural crest to the skin. Few cells can also be found in mucosae, eyes and leptomeninges, explaining primary extracutaneous localizations of melanomas.

Isolated LMNs are a challenging diagnosis in children because they are usually found in association to cutaneous melanomatosis. As previously reported, neurologic signs and symptoms of primary LMM are nonspecific, including seizures, psychiatric disturbances, and signs and symptoms of raised intracranial pressure, often with rapid evolution and fatal course [2]. Unlike most other cerebral tumors, the classic MRI appearance of LMM consists in high signal intensity on T1-weighted images and low signal intensity on T2-weighted images, depending on the presence of free paramagnetic radicals from melanin. Nonetheless, different signal patterns may be observed because of intratumoral hemorrhage [11]. A milestone in the characterization of cutaneous melanoma is the finding of the BRAF^V600E^ mutation in over 50 % of cases. Few molecular data are available about LMN making diagnosis challenging both on the clinical and pathological side [12].

Recent data suggest the presence of specific mutations in diffuse melanomatosis. Interestingly, different mutations have been found in adults (*GNAQ* and *GNA11* mutations) and children (NRAS^Q61K^), with BRAF^V600E^ mutation being observed in only 2 % of adult cases. In the largest series of children with cutaneous melanomatosis, 51 out of 66 were found to have the NRAS^Q61K^ mutation in their lesions [13]. Notably, in children with the neurocutaneous form (12 out of 16), the same mutation was found in leptomeningeal lesions suggesting a common origin of neoplastic precursors [14–16]. It has been suggested that a post-zygotic *NRAS* mutation of neural crest cells during embryogenesis, before migration to skin and leptomeninges, might condition a *NRAS* mosaicism in the same organism [17]. Nonetheless, mutations occurring before commitment to the neural crest lineage might explain the detection of the same *NRAS* mutation in tumors other than melanocytic. Interestingly, a NRAS^Q61R^ mutation has been reported in a spinal neurocristic hamartoma associated to NCM and leptomeningeal melanocytosis by Kinsler et al. Other observed tumors include meningioma and choroid plexus papilloma. Shih et al. reported a NRAS^G13R^ mutation in a primary mesenchimal brain neoplasm [18]. Invariably, the same mutation was documented in associated CMN but the observation of a germline single-nucleotide polymorphism of the MET gene suggests the possibility of a second hit to condition the clinical picture. In fact, *NRAS* mutations do not result in melanoma according to *in vitro* and *in vivo* preclinical models and to the evidence of mutated cells in CMN [19]. Possible co-operators in melanoma development include *MET* and *CDKN2A* [15, 20, 21].

Our case adds some information to the limited experience of the literature, confirming the presence of the NRAS^Q61^ mutation in children with meningeal melanomatosis. To our knowledge, this is the first case of LMN without associated skin lesions to harbor this mutation. Despite thorough clinical examination we cannot exclude the possibility of cutaneous melanoma having been overlooked in our patient. Nonetheless, cutaneous melanomas have also been described to regress spontaneously. Our child was initially treated for tuberculous meningitis based on MRI picture. LMNs have typically been described to show T1 hyperintensity and T2 hypointensity on baseline MRI [22]. Our patient did not show these features, making the diagnosis of infective meningitis more appealing in the first instance, even in presence of a negative work-up for TB. We would recommend reconsideration of diagnosis in case of suspect TB showing clinical-radiological progression during anti-tubercular treatment. Biopsy should be considered, not only to confirm diagnosis of LMN but also to study molecular profile and guide target therapy. *NRAS* inhibitors are not currently available but downstream pathways, such as MAPK and PI3K/AKT/mTOR constitute possible targets. In fact, promising results have been reported both in vitro and in vivo [16, 23–27]. Multi-target combinational approaches might help overcome resistance to treatment, however their clinical significance remains to be further determined [23, 24, 27–29].

Primary LMNs constitute a wide family of rare tumors. Peculiar pathogenetic mutations have been described in the adult (*GNAQ and GNA11*) and pediatric (*NRAS*) population. Most LMN present in association to cutaneous melanosis (NCM, CMN) and, in fact, a common molecular signature has been demonstrated in these cell populations. Our case is, to our knowledge, the first report of a LMN not associated to cutaneous findings but sharing the same NRAS^Q61^ mutation widely reported in the literature. Isolated LMN presentation might be insidious, mimicking TB meningitis, and should be suspected if no definite diagnosis is possible or if anti-TB treatment does not result in dramatic clinical improvement. Further molecular profiling and preclinical models will be pivotal in testing combination of target therapy to treat this challenging disease.

## Abbreviations

CSF, cerebrospinal fluid; LMM, leptomeningeal melanoma; LMN, leptomeningeal melanocytic neoplasms; MRI, magnetic resonance imaging; TB, tuberculosis
